# Combined Endovascular and Endoscopic Management of a Secondary Aortoesophageal Fistula after Open Surgical Aortic Repair in a Giant Descending Thoracic Aortic Pseudoaneurysm: Case Report and Review of Literature

**DOI:** 10.3390/jpm14060625

**Published:** 2024-06-11

**Authors:** Ovidiu Stiru, Reza Nayyerani, Mircea Robu, Roxana Carmen Geana, Petru Razvan Dragulescu, Oana Andreea Blibie, Serban-Ion Bubenek-Turconi, Vlad Anton Iliescu, Catalina Parasca

**Affiliations:** 1 Faculty of Medicine, Carol Davila University of Medicine and Pharmacy, 050474 Bucharest, Romania; ovidiu.stiru@umfcd.ro (O.S.); bubenek@alsys.ro (S.-I.B.-T.); vladanton.iliescu@gmail.com (V.A.I.); catalina.parasca@gmail.com (C.P.); 2 Department of Cardiac Surgery, Prof. Dr. C.C. Iliescu Emergency Institute for Cardiovascular Diseases, 022322 Bucharest, Romania; geana.roxana22@gmail.com (R.C.G.); razvan.dragulescu@ymail.com (P.R.D.); 3 1st Department of Cardiovascular Anesthesiology and Intensive Care, Prof. C. C. Iliescu Emergency Institute for Cardiovascular Diseases, 022328 Bucharest, Romania; blibieandreea@gmail.com

**Keywords:** secondary aortoesophageal fistula, TEVAR, esophageal stent, aortic repair

## Abstract

Secondary aortoesophageal fistula (AEF) is defined as a communication between the aorta and the esophagus, occurring after aortic disease treatment or esophageal procedures, associating very high mortality rates with treatment and being fatal without it. Several treatment strategies have been described in the literature, combining open surgery or endovascular aortic repair with surgical or endoscopic management of the esophageal lesion. We present the case of a 53-year-old patient with a history of open aortic surgery for a giant descending thoracic aortic pseudoaneurysm complicated with secondary AEF, successfully managed using emergency transiliac TEVAR (thoracic endovascular aortic repair), extensive antibiotic therapy associated with nutritional replenishment, and rehabilitation therapy. Novel endovascular and endoscopic devices have been developed, offering less invasive treatment strategies with improved outcomes, especially for high risk surgical patients. This case highlights the importance of a multidisciplinary approach to personalized medicine to manage such complex situations.

## 1. Introduction

Aortoesophageal fistula (AEF) is a rare entity of gastrointestinal hemorrhage associated with high mortality rates of over 77% with treatment and is fatal without any intervention [1,2]. It is defined as communication between the aorta and the esophagus, and it is classified as primary AEF and secondary AEF with an annual estimated incidence of 0.0015% and 0.6–2%, respectively [2,3]. Secondary AEF occurs after aortic disease treatment or esophageal procedures [4,5]. The incidence of secondary AEF after open thoracic aortic surgery is 1.7% [6,7,8].

Thoracic endovascular aortic repair (TEVAR) has become an alternative therapeutic approach for many thoracic aortic diseases, offering a less invasive alternative and lower postoperative morbidity rates compared to open surgery [1,6]. Emergency TEVAR can be used in the treatment strategies for AEF in order to achieve one of the mandatory conditions for survival: stopping exsanguination. However, the in-hospital mortality rate of TEVAR in patients with AEF ranges from 19% to 31% [4].

The pathogenesis of AEF is not well defined, but multiple mechanical and infectious factors are involved in its development [5]. AEF has a short clinical course and requires prompt diagnosis and emergency intervention [9]. The aortoesophageal syndrome in AEF was first described by Chiari as a sequence of dysphagia or mid-thoracic pain and sentinel minor bleeding expressed as minor hemoptysis or hematemesis, followed by a symptom-free period before a major bleeding event with exsanguination [3].

There is no consensus in the literature regarding the best treatment strategy for AEF, with multiple options available, including open surgery or endovascular aortic repair combined with management of the esophageal lesion (surgical or endoscopic) and extensive antibiotic therapy [1,3,7]. In this study, we present our approach for a complex case involving a high-risk and fragile patient. We successfully managed a secondary AEF in a 53-year-old patient with a history of open aortic surgery and esophagopleural fistula using emergency transiliac TEVAR, esophageal stenting, extensive antibiotic therapy associated with nutritional replenishment, and rehabilitation therapy. This case highlights the importance of a multidisciplinary approach to personalized medicine to manage such complex situations.

The patient signed an informed consent form for the publication of this report. This study was approved by the Institutional Ethics Committee.

## 2. Case Presentation

**Figure 1:**
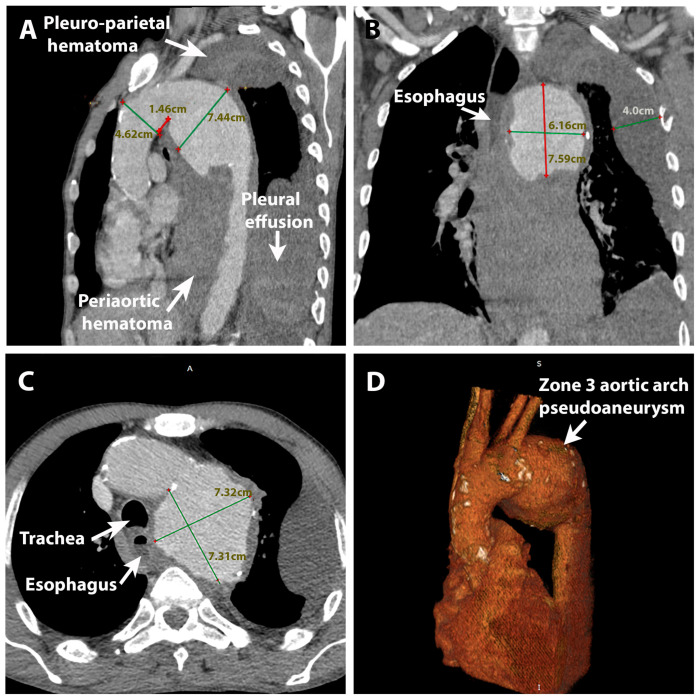
Preoperative angio-CT: (**A**) distal aortic arch pseudoaneurysm with periaortic hematoma, pleural effusion, and pleuro-parietal hematoma (sagittal plane); (**B**) (coronal plane); (**C**) (transverse plane); (**D**) angioCT reconstruction of the aorta with patchy atherosclerotic calcifications and distal arch pseudoaneurysm.

A 53-year-old male patient was admitted to our emergency department from a tertiary center with a diagnosis of severe anemia and minor hemoptysis. He was discharged from our institute 2 months prior to the current admission after open surgical aortic replacement for an aortic pseudoaneurysm in the isthmic region. At that time, he was diagnosed with a giant descending thoracic aortic pseudoaneurysm on computed tomography angiography (CTA) at another center after complaining of intense posterior thoracic pain and dyspnea. CTA revealed a 70 mm pseudoaneurysm at the isthmic level with periaortic hematoma. In addition, an image of a mediastinal hematoma with a compression effect on the esophagus was described. Left pleural effusion was also present, with additional dissection of the parietal pleura by the extensive periaortic hematoma. (Figure 1) The patient had multiple cardiovascular risk factors (systemic arterial hypertension, active smoking, age, and male sex) but no other significant medical history. Clinical evaluation revealed a cachectic patient (40 kg, BMI = 13.1 kg/m^2^) with dysphonia, general pallor, and the absence of a vesicular murmur on the left hemithorax. He was hemodynamically stable, with a BP of 100/50 mmHg, 80 bpm, and SpO2 of 98% in atmospheric air. Severe anemia was present, with a hemoglobin level of 7.9 g/dL. Echocardiographic findings were normal. Endovascular thoracic aortic repair (TEVAR) with partial debranching of the supra-aortic vessels was unsuitable because of the general dilatation of the ascending aorta and aortic arch with a diameter of 46 mm; thus, no proximal sealing zone could be achieved, even with the largest stent graft available. The pseudoaneurysm in the isthmic region was replaced with a Dacron Graft No. 32 (INTERGARD Woven Vascular Graft) via median sternotomy, left thoracotomy, right subclavian, and femoral cannulation. The proximal anastomosis was made on circulatory arrest with selective antegrade cerebral perfusion. The postoperative evolution was marked by sepsis of pulmonary origin, for which the patient received extensive antibiotic therapy combined with antifungal medication.

**Figure 2:**
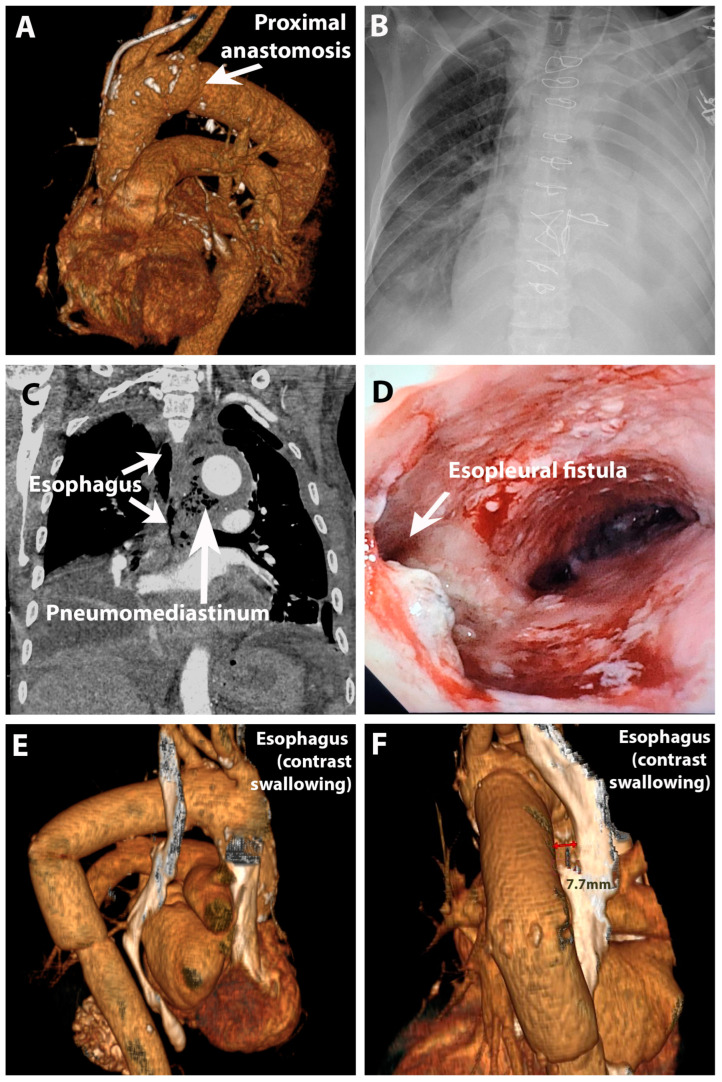
Postoperative imaging and esopleural fistula: (**A**) angioCT reconstruction of thoracic aorta after surgical distal aorta replacement; (**B**) Massive left pleural effusion; (**C**) Pneumomediastinum and esopleural fistula on angioCT; (**D**) Diagnosis of esopleural fistula on endoscopy before treatment with intraesophageal stent; (**E**,**F**): AngioCT reconstruction of aorta and esophagus (constrast swallowing) after treatment ((**E**)—posterior view; (**F**)—lateral view; aorto-esophageal distance 7.8 mm).

On the 14th day following surgery, a chest X-ray revealed the presence of a left pleural effusion and a collapsed left lung. (Figure 2B) Subsequently, a thoracostomy was performed. Milky pleural fluid was found, which yielded positive results in a color ingestion test using diluted methylene blue. A CT tomography revealed multiple lesions on the esophageal wall in the middle third of the left lateral wall, indicating a left esophageopleural fistula and an esophagobronchial fistula. (Figure 2C) Following a multidisciplinary meeting, the patient underwent esophageal stenting with a covered stent, followed by jejunostomy for enteral nutrition.

After multiple CT reassessments, we determined that the lesions had healed and the esophageal stent had been successfully removed. (Figure 2E,F) A follow-up endoscopy was performed for monitoring purposes, and the jejunostomy was subsequently removed, allowing the patient to gradually resume normal feeding. The patient was discharged after 100 days of hospitalization.

**Figure 3:**
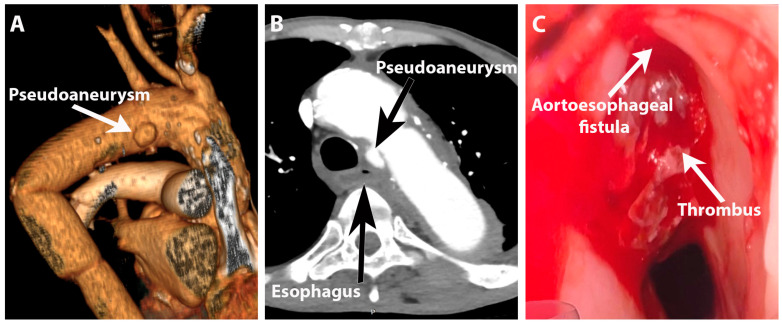
Secondary aortoesophageal fistula: (**A**) Aortic pseudoaneurysm at the level of proximal anastomosis (angioCT reconstruction, posterior view); (**B**) Aortic pseudoaneurysm and contact with the esophagus (transverse plane); (**C**) Endoscopic aspect of the aortoesophageal fistula on the anterior esophageal wall.

Two months after discharge, he presented to a tertiary center with mid-thoracic pain, dysphagia, minor hemoptysis, and fatigue. He was diagnosed with severe anemia (a hemoglobin level of 4.7 g/dL) and immediately transferred to our center. On admission, he was hemodynamically stable, with a BP of 105/75 mmHg, 75 bpm, and SPO2 of 100% in atmospheric air. He was admitted to having a low body weight of 37 kg and indicated that his diet consisted primarily of minimal fluid and semisolid food intake, with complete abstinence from meat and solid foods due to fear of recurrence of initial symptoms. He reported a few episodes of hemoptysis with a small amount of blood, with the last one being two days prior to the current admission. The patient did not notice any other changes and refrained from seeking medical attention until his general condition deteriorated. Severe anemia was corrected by the transfusion of 3 units of packed red blood cells, and the patient was transferred for CTA. Meanwhile, the patient developed massive melena. CTA revealed an aortic pseudoaneurysm (16/7.5 mm at the proximal anastomosis of the aortic graft) with possible communication with the left lateral wall of the esophagus but without noticeable contrast extravasation. CTA also revealed an 8 mm periaortic hematoma with gas bubbles situated between the esophagus and pseudoaneurysm. Another collection was identified around the distal anastomosis of the graft, which extended into the regional muscles and exhibited coastal lysis. The stomach contained heterogenic content suggestive of blood clots but no evident active source of bleeding (Figure 3A,B).

Following the CT evaluation, the patient had an episode of massive hematemesis (400 mL of fresh blood), became hypotensive, was immediately intubated for airway protection, and volemic resuscitation was initiated (blood product transfusion). Upper gastrointestinal endoscopy was performed using a standard endoscope with forward viewing, revealing a protrusion in the middle thoracic esophagus, 30 cm from the incisors, with an aspect of an old coagulum with red blood oozing from the middle and around the coagulum (Figure 3C).

**Figure 4:**
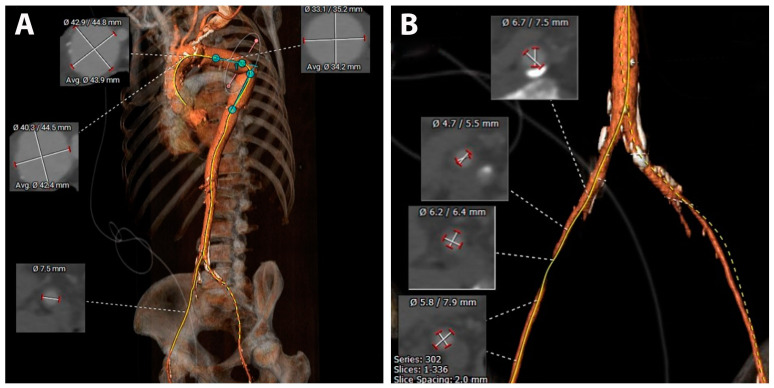
Pre TEVAR planning: (**A**) Assessment of the proximal landing zone and aortic arch anatomy; (**B**) Assessment of vascular access.

**Figure 5:**
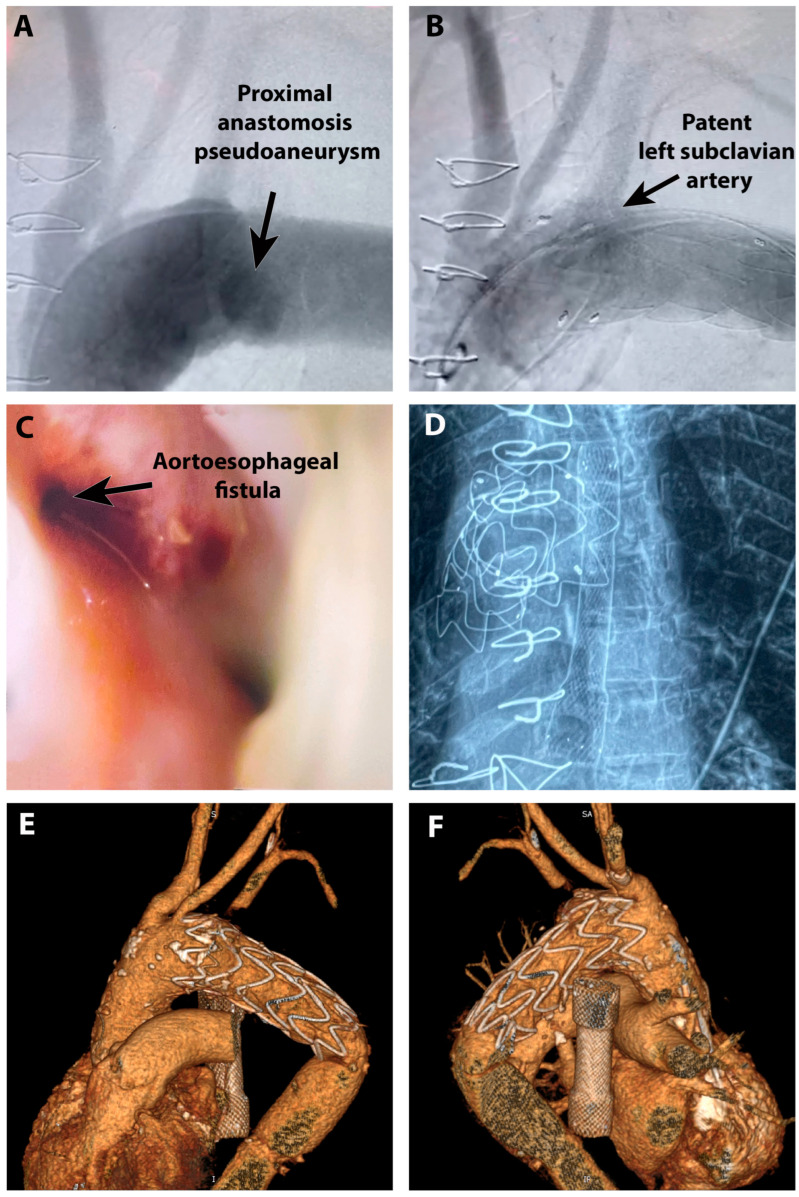
Treatment of secondary aortoesophageal fistula: (**A**) angiographic assessment of aortic arch anatomy and pseudoaneurysm; (**B**) Closure of pseudoaneurysm after TEVAR with coverage of left subclavian artery; (**C**) Endoscopic aspect of secondary aortoesophageal fistula after closure of pseudoaneurysm; (**D**) Angiographic aspect after esophageal stent implantation in relation to aortic stent; (**E**,**F**) angioCT reconstruction of aorta with closed pseudoaneurysm, left subclavian artery with retrograde filling from vertebral artery, in situ intraesophageal stent ((**E**)—anterior view); (**F**)—posterior view).

The diagnosis of secondary aortoesophageal fistula was confirmed, and taking into consideration all the details, we decided that emergency TEVAR as a life-saving procedure is the best option. 3D CT reconstructions were performed for preoperative planning, and we found that the diameter of the femoral vessels (7.5 mm) was insufficient for a 26Fr sheath (Figure 4). We decided to use a transiliac approach; therefore, the intervention started with a longitudinal incision in the right abdominal flank. The retroperitoneal space was entered, and the iliac vessels were isolated. The common right iliac artery was considered suitable for vascular access, and we proceeded with the introduction of the sheath and guidewires. A 46 mm diameter by 100 mm long thoracic stent-graft Valiant Thoracic Endograft with the Captivia Delivery System (Medtronic Vascular, Santa Rosa, CA, USA) was positioned and deployed in the first portion of the descending thoracic aorta. The stent graft was further expanded in the proximal landing zone by post-dilatation using a Reliant balloon. Postprocedural aortic arch angiography demonstrated total exclusion of the pseudoaneurysm with no evidence of endo-leaks (Figure 5A,B).

The patient’s condition remained stable following the surgical procedure. Consequently, on the second postoperative day, we initiated the treatment of the esophageal lesion using a fully covered esophageal stent (Cook Evolution 80/20/25) (Figure 5C,D). Furthermore, a nasogastric tube was inserted to establish a feeding route.

A CTA re-evaluation was performed 1 month postoperatively, suggesting no esophageal leakage or graft infection (Figure 5E,F). The patient followed a slow, favorable postoperative evolution and was introduced into a rehabilitation program for functional recovery and weight gain.

## 3. Review and Discussion

### 3.1. Etiology and Pathogenesis 

AEF are classified as primary (0.0015%) and secondary (0.6–2%) [2,3]. Primary AEF is mostly caused by thoracic aortic aneurysms (51%), while other causes include penetrating aortic ulcer, esophageal cancer, ingestion of foreign bodies, Barrett’s ulcer, trauma, or prolonged nasogastric tube intubation [4,5]. Secondary AEF occurs after the treatment of aortic disease (surgical or endovascular interventions) or after esophageal procedures (endoscopic or operative) [4,5].

The pathogenesis of AEF is not well defined, but multiple mechanical and infectious factors are involved in its development [5]. Several studies have described the multiple pathophysiological mechanisms involved in the development of AEF:

(1) Esophageal ischemia is caused by compression from a large aortic aneurysm or pseudoaneurysm, followed by loss of the aortic side branches that feed the esophagus after open surgical repair or covered by a stent graft in endovascular treatment [9,10];
(2) The presence and resorption of periaortic hematoma trigger an aggressive regional inflammatory response, changing the histopathological structure of the esophageal wall. This is followed by the occurrence of adhesions, tissue necrosis, erosion, and fistulous communications [6,9]. Chiesa R and his colleagues admit that these modifications (local inflammatory response and surrounding compression) are not always completely ameliorated, even after TEVAR; thus, they are involved in the development of late fistulization [6];
(3) Mechanical damage in the affected region is caused by continuous arterial pulsation of a “modified” aortic wall. And when we say “modified” aortic wall, we mean either the synthetic material used for aortic replacement in surgical approaches (Ex. Dacron) or the rigidity of the aorta and the geometrical changes induced by the endoprostheses. (Radial force of the stent graft against the native wall) [1,10].
(4) Primary infection of the aneurysmal wall may be a potential cause of primary AEF, and infection of the aortic replacement graft or stent graft can be considered the main mechanism of secondary AEF, as Xi et al. suggested [6,10].

In our case, risk factors for AEF were the following: first compression of the pseudoaneurysm caused esophageal ischemia, and this was exacerbated by the loss of esophageal vascularization after the first surgery; furthermore, the inflammation in this area both from surgery and from the resorption of the periaortic hematoma affected the esophageal wall; local infection after AEF occurrence produced a vicious circle, further damaging the esophageal wall. We could not find any guidelines for monitoring AEF formation in this kind of patient. Considering the experience in this case, daily clinical evaluation for symptoms such as dysphagia, hemostasia/melena, and thoracic pain, daily chest XR’s, and close monitoring of inflammatory markers and hemoglobin levels could help raise suspicion for AEF formation. Also, a follow up CT scan in the immediate postoperative period, especially in patients with large descending aorta pseudoaneurysms, could be a valuable tool in the diagnostic and prevention of AEF. 

Regarding AEF after TEVAR, Eggebrecht et al. described some pathophysiologic mechanisms: (1) direct erosion of the aortic wall and the esophageal wall due to rigid endoprostheses; (2) pressure necrosis of the esophageal wall caused by continuing forces of the self-expanding stent-graft; and (3) infection of the endoprosthesis [6,10].

### 3.2. Clinical Presentation

Aortoesophageal syndrome in AEF was first described by Chiari in 1914 as a sequence of dysphagia or mid-thoracic pain, sentinel minor bleeding expressed as minor hemoptysis or hematemesis, followed by a symptom-free period before a major bleeding event with exsanguination.(1,3,10) However, in a review of the literature consisting of more than 500 cases, only 45% of the patients presented with classical Chiari’s triad, while herald bleeding was found in 65% of the cases, mid-thoracic pain in 59%, and dysphagia in 45% of the cases [5]. Another study identified hematemesis as the most common symptom of AEF [1].

Our case involved a patient who experienced dysphagia; however, it is essential to consider a psychogenic component, as the patient admitted to being fearful of potential complications associated with eating. On anamnesis, we discovered that he avoided consuming meat, leading us to suspect that the lack of protein in his diet may have contributed to his condition. The patient also reported a few instances of minimal hemoptysis and hematemesis, with the most recent episode occurring 48 h prior to admission to our facility. However, this was followed by significant episodes of melena and hematemesis, indicating the presence of the classical Chiari’s triad in this case.

Jonker et al. described that in their study, 95% of their patients had an acute presentation, and hypovolemic shock and systemic infection were present in 33% and 36%, respectively [10]. Our patient had an acute presentation but was initially stable after the occurrence of hypovolemic shock, an episode of massive hematemesis.

### 3.3. Imagistic Evaluation

Several diagnostic tools are available. Supine chest RX can be a basic screening investigation, but the most sensitive and specific is endoscopy, as it can offer a clear view of the lesion [6]. Several forms of AEF have been described in the literature: submucosal tumor-like protrusion, ulcerative lesions with or without active bleeding, pulsating protrusions with central fistula, bleeding only, or even direct view of the stent-graft in cases of secondary AEF after TEVAR [1]. Endoscopy can also describe the location of the lesion in the esophagus, providing anatomical landmarks along the surrounding organs. The majority of AEF is located between the posterior wall and the left lateral wall of the esophagus, as in our case [1]. We found a protrusion with an aspect of an old coagulum with red blood oozing from the middle and around the coagulum in the middle thoracic esophagus. 

According to some studies, repeated endoscopic evaluations may constitute a risk factor for aortic aneurysm ruptures [1]. They also recommend trans-nasal small-caliber endoscopy as a safer procedure [1].

Yamazato et al. suggest the use of endoluminal echo esophagography to evaluate the thickness of the esophageal wall [7]. 

High-resolution CT can be used to evaluate the aorta, the presence of periaortic hematoma, signs of mediastinitis such as air bubbles in the thrombus, around the aortic graft or esophagus, and esophageal wall abnormalities. However, it can rarely directly identify fistulous communication, as the majority of these are low-flow. In most of the cases, as it was in ours, the CT evaluation revealed indirect signs of the AEF. Here, we had a proximal anastomosis pseudoaneurysm in contact with a thickened left lateral esophageal wall, with a collection of air bubbles in the mediastinum. Another collection was identified around the distal anastomosis associated with costal lysis. These findings suggest mediastinitis or infection of the aortic graft, even though the patient did not present any signs of systemic infection (fever or shivers, low WBC count), and he also received an extensive spectrum of long-term antibiotic therapy. CT can also be useful in therapeutic management for drainage of the mediastinal collection and identification of the pathogenic agent, but this procedure was not available in our center [6].

### 3.4. Treatment Strategies

With the development of endovascular techniques, an increasing number of patients benefit from TEVAR for aneurysmal diseases. In fragile patients with multiple comorbidities like ours, endovascular repair is a feasible option with better postoperative outcomes and reduced mortality compared to open surgery. However, our patient was not suitable for TEVAR for his initial pseudoaneurysm due to severe atherosclerosis and dilatation of the ascending aorta and aortic arch (46 mm), which was incompatible even with the largest stent graft available. Therefore, we performed a surgical replacement of the aorta. However, these procedures can result in several complications. Secondary AEF has an incidence of 1.7% after open aortic surgery [6,7,8]. AEF treatment aims to achieve two primary objectives: prompt hemostasis and fistula closure.

Several surgical strategies have been reported in the literature, but even in centers of excellence, operative mortality can reach more than 50% due to the emergent nature of the intervention, redo operation, difficulties in accessing the aorta surrounded by hematoma, difficulties in cross-clamping, adhesions with significant blood loss, possible mediastinitis, and the risk of visceral injury [4,7,11,12]. Multiple studies in the literature suggest removing the infected aortic graft and replacing the aorta after the mediastinal collection is debrided, in combination with single- or dual-stage repair of the esophagus, but only if the patient’s condition permits such complex surgical procedures [6]. This was not possible in our situation, considering the cumulated comorbidities and the risks of a redo operation in fragile patients with herald hemorrhage [6]. But there are also several cases successfully treated without removing the graft using modern, available technologies. Therefore, we opted for a less invasive procedure and evaluated the opportunity for lifesaving TEVAR. 

In a systematic review by Canaud et al., 83.3% of the evaluated patients received TEVAR within the first 24 h after diagnosis, with a technical success rate of 87.3% and an overall 30-day mortality rate of 19.4% [12]. In a published meta-analysis, in-hospital mortality for TEVAR after AEF was between 19% and 31% compared to operative mortality after open surgery in AEF, which was more than 50% [4,10]. In the same study conducted by Canaud et al., the most common early complication was persistent postoperative sepsis (23.6%), and we found this also in our case [12]. Other complications that may appear after TEVAR for AEF are aortic rupture (5.5%) due to an inflammatory tissue, a possible infected region, extensive atherosclerosis combined with the rigidity of the stent graft, endoleak (9.7%), or recurrent hematemesis (5.5%) [12]. There have also been reported cases of secondary AEF after TEVAR [7].

The use of TEVAR in this case was also challenging. First, we faced a modified aorta. Histologically, the evaluation during the first procedure showed that the aorta was full of atherosclerosis and calcar plaques. This was associated with a massive inflammatory response of the native aorta and infection of the aortic graft. On the one hand, these were possibly factors involved in the appearance of the pseudoaneurysm of the proximal anastomosis; on the other hand, all these factors increased the risk of intraprocedural rupture of the vulnerable aorta. Second, we encountered technical difficulties regarding vascular access. Femoral vessels were not suitable for stent graft delivery because of their small diameter and severe atherosclerosis. Other access sites include the transiliac approach (via the common iliac arteries) or transaortic approach (via the infrarenal aorta), in a direct manner, or using the conduit technique. Another important aspect was obtaining a sufficient proximal sealing zone. The distance between the pseudoaneurysm and the origin of the left subclavian artery was less than 2 cm, as recommended by the manufacturer. Implantation of the stent graft required, in our case, coverage of the left subclavian artery to achieve an adequate proximal landing zone. Revascularization of the upper arm was taken into consideration (left carotid artery to left subclavian artery bypass), but it was not necessary in our case, as adequate perfusion of the arm was still present through the collateral vessels. 

Moreover, the anatomy of the aorta was modified by the initial pseudoaneurysm, followed by the first open surgical procedure. These modifications increase the difficulty of stent graft deployment, with the risk of malposition or even graft migration. 

The second major goal in the treatment of AEF is to treat the esophageal lesions. Several surgical options have been described with single- or dual-stage interventions [13]. Those techniques include lateral cervical esophagostomy, jejunostomy, or gastrostomy for the feeding route and evacuation of the mediastinal collection, followed by reconstruction of the esophagus with jejunum interposition or gastroplasty [14]. Other authors have performed direct suturing of esophageal lesions and omentoplasty [15,16]. Pericardial, pleural, or intercostal muscle flaps can be used for covering [12]. Rodrigues-Pinto et al. suggest that surgical repair of the esophagus should be made in stable patients when their general condition is suitable for multiple surgical procedures since the esophageal reconstruction carries out an increased surgical trauma [13]. They also propose the use of esophageal stents as a less invasive and equally effective treatment [13]. The stents bypass the esophageal defect, allowing it to heal. Depending on the stent, oral feeding can be quickly resumed, or a fasting period may be necessary [8]. Nutritional replenishment is a key factor in recovery. Caloric balance and macronutrients can be easily calculated and adapted for each individual case. These can be achieved by enteral feeding through a gastrostomy, jejunostomy, or nasogastric tube, or by parenteral feeding, with various products being available. In our case, we used a novel esophageal stent that required only 3–5 days of nasogastric or parenteral feeding.

In a study conducted by Canaud et al., patients who underwent TEVAR with concomitant adjunctive procedures (esophageal repair, resection, or stenting) had lower aortic-related mortality rates [12].

As a treatment strategy, it is mandatory to include extensive empirical broad-spectrum antibiotics associated with antifungal medication. In most cases, antibiotics are administered for a prolonged period (>4 weeks) [12].

The treatment of AEF is complex and requires a multidisciplinary approach, as most cases require emergent life-saving solutions during the acute phase. Table 1 summarizes the main studies regarding the management of AEF. In addition to the high mortality rate associated with this pathology, several successful cases have been reported. Long-term follow-up and lifestyle control are important during the postoperative period. Another factor that can improve the results is cardiac rehabilitation (CR). The 2013 Canadian Cardiovascular Society guidelines mentioned CR for the first time as a safe treatment in patients following thoracic aortic repair, with the potential to reduce mortality [17,18]. Physically active behavior and regular exercise are also recommended by the latest AHA and ESC guidelines on the management of aortic disease. Several studies have concluded that CR should be an integrated part of postoperative management [19,20]. In the Aneurysm CaRe study conducted by Bahia SS et al., it was shown that CR can improve cardiovascular function and physical recovery, making faster socio-professional reintegration possible and increasing the quality of life [21]. In our case, the patient entered an intensive rehabilitation program considering his poor physical condition from a long period of enteral feeding and immobilization in the intensive care unit. We recommended serial chest CT scans at 3 months, 6 months, and one year. Also, an upper digestive endoscopy should be performed at 3 months to evaluate the possibility of esophageal stent extraction. The rationale for serial chest CT scans, beyond the obvious control of the aorta and esophageal status, should also be the diagnosis of other possible aortic pseudoaneurysms that could further produce AEF’s.

## 4. Conclusions

The exponential development of technology offers less invasive treatment strategies for high-risk patients. Novel endovascular devices are available, offering lower physiological impact and improving outcomes by reducing operative stress and avoiding surgery-associated complications. Despite all of those, further improvements in the endovascular area should be made for a wider spectrum of aortic arch pathologies, which are now only covered by surgery. Few survivors have been reported in the literature, once again emphasizing the high mortality rates associated with this pathology. Early detection is required; therefore, active screening after major aortic surgery for this rare but possible complication is advised.

Further studies should be made to identify statistically significant associated risk factors or possible predisposing factors for AEF. We encourage a multidisciplinary approach and a multistage treatment strategy for such a complex pathology.

## Figures and Tables

**Table 1:** Main studies regarding the management of AEF (AEF: aorto-esophageal fistula; NS: not specified; TEVAR: thoracic endovascular aortic repair; SAR: surgical aortic replacement).

Authors	Year	Country	Number of Patients	Age (Years)	Type of AEF	Management of Aortic Lesion	Treatment of AEF	Follow-Up	Mortality
Enomoto et al. [11]	2020	Japan	4	80 ± 8	Secondary	1 case of TEVAR and 1 case of SAR	Esophagectomy and esophageal reconstruction	NS	25%
Chiba et al. [1]	2013	Japan	1	70	Secondary	SAR	Omental implantation	10 months	0%
Jonker et al. [10]	2009	Netherlands	43	62 ± 2.5	NS	TEVAR	Surgical	5 months	32.5%
Monteiro [2]	2020	Portugal	1	41	Primary	TEVAR	Endoscopic clips	Ns	Ns
Canaud et al. [12]	2014	England	72	59.2	26 Primary 46 secondary	TEVAR	Surgical	7.4 months	40.2%
Yamazato et al. [7]	2017	Japan	18	67.2 ± 10.4	Secondary	3 TEVAR 3 bridge TEVAR to open surgery 12 SAR	Surgical Resection and omental flap	60 months	57.6%
Rodrigues-Pinto [13]	2015	Portugal	1	63	Secondary	TEVAR	Esophageal Stent	NS	NS
Wu et al. [3]	2022	China	1	58	Secondary	TEVAR	Esophageal Stent	6 months	0%
Furui et al. [8]	2021	Japan	1	72	Secondary	TEVAR	Surgical, muscle flap and Esophageal Stent	NS	NS
Yuhara [15]	2017	Japan	1	77	Secondary	SAR	Omental flap	36 months	0%

## Data Availability

Data is available on request.
